# Efficacy of Bimanual Training With Lower-Limb Activities on Attention, Coordination, and Concentration in Children With ADHD

**DOI:** 10.7759/cureus.111394

**Published:** 2026-06-23

**Authors:** Dipti D Kadam, Mandar Malawade

**Affiliations:** 1 Department of Pediatric Neurosciences Physiotherapy, Krishna College of Physiotherapy, Krishna Vishwa Vidyapeeth (Deemed to be University), Karad, IND

**Keywords:** attention, attention deficit hyperactivity disorder, bimanual training, bot-2, concentration, conners parent rating scale, lower limb, motor coordination, pediatric physiotherapy

## Abstract

Introduction

Attention-deficit/hyperactivity disorder (ADHD) is a prevalent neurodevelopmental disorder in children, marked by inattention, hyperactivity, impulsivity, and impaired motor coordination. While pharmacological and behavioral interventions remain the primary management strategies, growing evidence highlights the influence of motor-based interventions on cognitive and attentional functions. Physiotherapy approaches incorporating bimanual and whole-body movements may enhance sensory integration, bilateral coordination, and executive control. However, limited research exists on structured play-based bimanual training combined with lower-limb activities in children with ADHD.

Method

An experimental pre-post interventional study was conducted at the Department of Pediatric Physiotherapy, Krishna College of Physiotherapy, Krishna Vishwa Vidyapeeth (KVV), Karad, and Chaitanya Bal Rugnalaya, Karad. Eighteen children aged 6-12 years with mild to moderate ADHD were recruited through convenience sampling. Participants underwent a 12-week play-based bimanual training program with lower-limb activities using hopscotch mats, sensory boards, balance trainers, Swiss balls, and sorting tasks. Motor proficiency and behavioral outcomes were assessed pre- and post-intervention using the Bruininks-Oseretsky Test of Motor Proficiency-2, Second Edition (BOT-2) (Short Form) and Conners’ Parent Rating Scale-Revised (CPRS-R).

Result

Results showed statistically significant improvement in motor coordination, attention, and concentration following the 12-week intervention. BOT-2 scores improved significantly from 32.17 ± 3.35 to 41.11 ± 3.08, while CPRS-R scores reduced from 160.61 ± 25.39 to 105.56 ± 14.42, indicating decreased ADHD symptom severity and enhanced behavioral performance.

Conclusion

Bimanual training with lower-limb assistance is an effective, engaging physiotherapy intervention for children with ADHD. It simultaneously addresses motor coordination deficits and cognitive-behavioral deficits, making it a valuable adjunct to existing ADHD management strategies. Future large-scale randomized controlled trials are recommended to further establish its clinical efficacy.

## Introduction

Attention-deficit/hyperactivity disorder (ADHD) is listed under neurodevelopmental disorders in the Diagnostic and Statistical Manual of Mental Disorders, 5th Edition (DSM-5) and is characterized by behavioral symptoms that manifest early in childhood and across multiple contexts such as home, school, and play, reflecting a continuous pattern of inattention or hyperactivity-impulsivity that impairs functioning or development [1]. Cognitive, behavioral, and psychosocial functioning are all impacted by the illness, which usually first appears before age 12 and may continue throughout adolescence and adulthood [2]. Children with ADHD show deficits in working memory, inhibitory control, cognitive flexibility, planning, attention management, executive functioning, and behavioral control, which affect everyday, social, and academic functioning [3]. ADHD symptoms typically emerge in early childhood and are most commonly identified during the early school-age years, between six and nine years of age, when increasing academic and behavioral demands make symptoms more apparent to parents and teachers, and according to longitudinal research, almost 60% of children with ADHD continue to show symptoms into adolescence and adulthood [4].

With a male-to-female ratio of 2:1 to 4:1 and a global incidence of around 5%-7% among school-age children, ADHD is a major public health issue, more commonly diagnosed in boys, who tend to show hyperactive-impulsive symptoms, while girls more often show inattentive symptoms [5]. Its high heritability reflects a multifactorial etiology involving genetic, neurological, and environmental factors, including prenatal toxin exposure, low birth weight, and psychosocial stressors [6]. Functional imaging studies show reduced activity in frontal brain regions, particularly the prefrontal cortex, during inhibitory control and attention tasks in children with ADHD [5], while anatomical and functional cerebellar defects-given the cerebellum's role in motor coordination, timing, and cognitive processing-further worsen motor and attentional deficits [7].

Cognitive and motor symptoms substantially affect day-to-day functioning: inattention, distractibility, and difficulty completing tasks; hyperactivity and restlessness; and impulsivity, all of which interfere with academic and social performance. Related impairments in motor coordination and planning-deficits in fine and gross motor skills, balance, and bilateral coordination-result in clumsiness, poor handwriting, difficulty throwing or catching, and reduced involvement in sports [8,9]. Attention involves selecting and concentrating on relevant inputs, while concentration involves sustaining focus on goal-directed tasks over time [10]. Inadequate attentional control can impair motor coordination and task performance by limiting sensory feedback integration, leading to slower motor learning and reduced accuracy in coordinated activities [7,11]. Motor coordination activities require sensory integration, motor planning, and cognitive control, and when attention is disrupted, motor coordination is affected, causing clumsiness and reduced motor proficiency [12]. Children with ADHD often show difficulties with bilateral coordination, motor planning, and inhibitory control needed for complex motor sequencing [13]. Coordinated motor activity can benefit cognitive functioning, as the cerebellum and prefrontal cortex-regions with overlapping neural networks for motor coordination and attentional processes-are often dysfunctional in ADHD, impairing coordination, attention, and concentration [8,14]. Bimanual coordination, the purposeful and precise use of both hands together for tasks such as writing, dressing, catching, and tool use, is often trained to improve coordination during rehabilitation; children with ADHD frequently show bimanual coordination difficulties with implications for everyday activities and play, reflecting deficits in motor planning, timing, and interhemispheric communication [15].

Repeated practice of coordinated upper-limb movements can improve coordination through neural restructuring [16]. Play-based treatment offers an engaging, child-centered environment that increases motivation, engagement, and motor skill practice, supporting interhemispheric communication and effective movement patterns while enhancing attention, concentration, coordinated movement, social skills, and problem-solving [17,18]. Building on principles of motor learning, repetition, and goal-directed training, Hand-Arm Bimanual Intensive Therapy Including Lower Extremities (HABIT-ILE) is an intensive rehabilitation strategy that encourages bilateral use of the hands and lower limbs to enhance motor performance and functional independence in children with neurological conditions [19]. Intense motor practice can enhance focus, coordination, and attention by strengthening neural pathways involved in motor control and inducing neuroplastic brain changes [16]; the repetition, meaningfulness, and motor demands of such tasks activate neural networks required for motor and cognitive function, and childhood neuroplasticity allows considerable developmental change through therapeutic intervention [8]. Combining play activities with bimanual coordination training may therefore offer a comprehensive approach to motor and cognitive impairments in ADHD [17]. While previous studies have shown favorable effects of physical activity, motor training, and play-based interventions in children with ADHD, few have examined the combined effects of bimanual coordination training involving the lower limbs on attention, coordination, and concentration, and research on structured bimanual training within play-based ADHD therapy remains limited [17]. Previous studies have shown the favorable impact of physical activity, motor training, and play-based interventions on children demonstrating ADHD, but few studies have focused on the combined effects of bimanual coordination training that engages the lower limbs on attention, coordination, and concentration outcomes. There is currently limited research on structured bimanual training programs as part of play-based therapeutic activities for ADHD populations [17], and previous literature indicates that coordinated motor activities can shape cognitive aspects like attention and executive control. Most existing motor-based interventions for ADHD have relied on either isolated upper-limb tasks or general gross motor/aerobic activity, which may not adequately engage the interhemispheric and cerebellar-prefrontal networks that underlie both motor coordination and attentional regulation. Bimanual training combined with lower-limb activity is expected to provide additional benefit over these standalone approaches because it simultaneously demands interlimb timing, whole-body postural control, and vestibular-proprioceptive integration, placing greater demand on the same overlapping neural circuits implicated in attention and executive function. Additionally, the majority of studies have concentrated on pharmacological or behavioral interventions, and relatively little attention has been paid to motor-based therapeutic interventions that target the upper and lower limbs concurrently. Given the close ties among motor coordination, attention, and concentration, an intervention that integrates bimanual upper-limb tasks with lower-limb and whole-body coordination could therefore have more holistic and synergistic effects on children with ADHD than interventions addressing these systems in isolation. Therefore, this study aimed to investigate the effects of bimanual training involving lower limbs on attention, coordination, and concentration of children with ADHD.

## Materials and methods

An experimental pre-post interventional study was conducted at the Department of Pediatric Physiotherapy, Krishna College of Physiotherapy, Krishna Vishwa Vidyapeeth (Deemed to be University), Karad, and at Chaitanya Bal Rugnalaya, Karad, Maharashtra, India. Ethical clearance was granted by the Institutional Ethics Committee of Krishna Vishwa Vidyapeeth (Protocol No. KVV/IEC/10/2025) on November 5, 2025. Informed written consent was obtained from parents or legal guardians of all participants before enrolment, and written assent was taken from children aged six years and above, ensuring voluntary participation.

Participants

Sample size was evaluated using the formula $n=Z^{2}\cdot p\cdot q/L^{2}$, where *p* = 7.1% (ADHD prevalence), *q* = 100 − *p*, *Z* = 1.96 (95% confidence level), and *L* = 12% (margin of error), yielding a calculated minimum sample of *n* = 18. Participants were recruited by convenience sampling over one year.

The study included 18 children diagnosed with ADHD aged between six and 12 years, with a mean age of 7.56 ± 1.85 years. The sample showed a predominantly male distribution, comprising 12 males (66.67%) and six females (33.33%), which corresponds to a higher prevalence of ADHD reported in males in existing literature. The largest age subgroup was six years (44.44%), indicating variability in age distribution and reflecting the heterogeneous clinical presentation of ADHD among school-going children.

Inclusion and exclusion criteria

Children aged 6-12 years, of either sex, who were currently enrolled in mainstream schooling and had a pre-existing diagnosis of mild to moderate ADHD established by a pediatrician, child psychologist, or developmental pediatrician using standardized clinical assessment tools DSM-5 diagnostic criteria and the Vanderbilt ADHD Diagnostic Rating Scale, and who were able to understand and follow instructions for the intervention and assessments, were included in the study. The severity classification (mild to moderate) was determined by the referring clinician at the time of diagnosis and was not re-assessed by the study team. The same inclusion criteria were applied uniformly to male and female participants, with no sex-specific diagnostic thresholds used; sex was instead recorded as a demographic variable, given the known differences in symptom presentation between boys and girls with ADHD. Children with coexisting neurological, musculoskeletal, cardiorespiratory, or other developmental disorders such as intellectual disability or cerebral palsy, as well as those with severe visual, auditory, or speech disabilities, were excluded from the study.

Outcome measures

The Bruininks-Oseretsky Test of Motor Proficiency, Second Edition (BOT-2) (Short Form) was used to assess fine and gross motor skills across eight subtests, including balance, bilateral coordination, strength, and manual dexterity, applicable to children aged 4-21 years.

Conners' Parent Rating Scale-Revised (CPRS-R) was used to assess ADHD symptoms and related behavioral outcomes, including inattention, concentration, hyperactivity, and impulsivity. The CPRS-R demonstrates high internal consistency (α = 0.85-0.95), strong test-retest reliability, and excellent construct and criterion validity aligned with DSM-5 criteria [20]. Prior to beginning the intervention, comprehensive pre-assessment evaluations were conducted to establish baseline measures of the dependent variables. Pre-assessment included the following: (1) Evaluation of attention and concentration using the total score of the CPRS-R scale, with parents completing the rating scale based on their observations of the child's behavior at home and in community settings. (2) Assessment of hand-eye coordination, manual coordination, and balance using relevant subtests from the BOT-2. (3) Collection of demographic and clinical information. These baseline measures served as a baseline for comparison with post-intervention scores to evaluate the magnitude of change.

Intervention protocol

The intervention was delivered over 12 weeks, consisting of four sessions per week lasting 30 to 45 minutes, including structured rest intervals of approximately 2-3 minutes between each activity set, with a maximum cumulative rest period of 10 minutes per session, designed to prevent fatigue-related performance decrements while maintaining the child's engagement and cognitive alertness throughout the session. This frequency and duration were chosen based on evidence suggesting that intensive motor training, typically defined as frequent weekly sessions administered across several weeks, promotes neuroplasticity and strengthens neural pathways for motor control and attention. The duration of individual sessions was designed to challenge the child cognitively and physically while preventing fatigue-related decrements in performance and maintaining engagement.

The intervention incorporated multiple evidence-based motor and cognitive activities organized into six categories.

Motor Orientation Activities

These foundational activities included the rainbow sorting task and Jack & James sensory board activities, performed in two to three sets with five to six items progressing to 10 to 12 items as the child's performance improved. These activities targeted color recognition, attention, and eye-hand coordination, providing a gentle introduction to the intervention and building foundational skills [17,18].

Bilateral Coordination Activities

The cloud hopscotch mat and arm and foot hopscotch equipment were used to perform three to four sets of full mat sequences. These activities were designed to improve coordination between limbs, develop rhythm awareness, and enhance balance through tasks requiring simultaneous movement of upper and lower extremities in coordinated patterns [15,16].

Cognitive-Motor Integration

Gisco Sports soccer mini disc cones were used in three to four sets to work on divided attention, sequencing, and goal-directed movement. These activities required children to track moving objects, plan movement sequences, and make rapid decisions, thereby engaging both cognitive and motor systems simultaneously [11].

Swiss Ball Therapy

This component included seated balance activities, prone reaching exercises, and bouncing tasks performed for three to five repetitions. Swiss ball training improves core control, postural stability, and proprioceptive awareness-all of which contribute to better attention and body awareness [8].

Sensory Integration Therapy

A sensory mat with different textures was used for three to four trials per texture type, providing tactile and proprioceptive input that helps children presenting sensory processing difficulties regulate their nervous systems and improve focus [21].

Vestibular Training

This included trampoline jumping (three to 10 repetitions for 20 s each), half-sphere balance board training, and swing therapy (two to three repetitions for five minutes each). These activities stimulate the vestibular system, which is crucial for balance, postural control, and attention regulation-areas where children affected by ADHD often show deficits [7,21].

Intensity and complexity progression

The detailed intensity and complexity progression protocol followed throughout the intervention period, including the gradual advancement of task difficulty and activity levels across different training phases, is presented in Table 1.

**Table 1:** Intensity and complexity progression This frequency and duration were selected based on evidence from systematic reviews demonstrating that structured motor interventions administered over 8-12 weeks at 3-5 sessions per week promote neuroplasticity, strengthen neural pathways for motor control and attention, and produce significant improvements in motor proficiency and behavioral outcomes in children with ADHD [16,22,23] ADHD: attention-deficit/hyperactivity disorder

Activity category	Phase I (Weeks 1-4): low complexity	Phase II (Weeks 5-8): moderate complexity	Phase III (Weeks 9-12): high complexity
Motor orientation activities: rainbow sorting task, Jack & James sensory board	Use one color and one bowl, sort 5-6 items; simple matching on sensory board.	Use two colors and two bowls, sort 8-10 items; faster matching on sensory board.	Use multiple colors (3-4 bowls), sort 10-12 items; increase speed and accuracy.
Bilateral coordination activities: cloud hopscotch mat, arm & foot hopscotch	Perform basic hopscotch pattern, slowly focus on correct hand-foot placement.	Perform alternating hand-foot patterns across the mat, increase rhythm and coordination.	Perform continuous full mat sequence, increase movement speed and coordination.
Cognitive-motor integration: Gisco Sports soccer mini disc cones	Arrange cones in straight line pattern, simple movement between cones.	Arrange cones in different directional patterns, follow movement sequence.	Arrange cones in complex patterns, quick directional changes and sequencing.
Swiss ball therapy	Perform seated balance and gentle bouncing (3-5 repetitions).	Perform prone reaching and controlled bouncing.	Perform dynamic balance with reaching and bouncing.
Sensory integration therapy: sensory mat	Walk on one texture at a time.	Walk on two to three textures sequentially.	Walk on multiple textures continuously.
Vestibular training: trampoline, half-sphere balance board, swing therapy	Perform basic trampoline jumping, supported balance board standing, and gentle swinging.	Perform controlled jumping and independent balance board standing.	Perform repetitive jumping, dynamic balance board tasks, and coordinated swinging.

The intervention protocol was developed by synthesizing these individually evidence-based motor, sensory, and vestibular components into a structured, progressive, play-based framework, conceptually informed by the HABIT-ILE model. While the protocol document has been filed for copyright protection (Application No. LD-15193/2026-CO), it has not yet undergone formal content validity assessment or independent feasibility testing outside this study.

## Results

All 18 children enrolled in the study completed the full 12-week intervention protocol with no dropouts or discontinuation of treatment sessions; consequently, pre- and post-intervention data were available for all 18 participants (n = 18) for both outcome measures.

All statistical analyses were performed using IBM Statistical Package for Social Sciences (SPSS) software version 25.0 (IBM Corp., Armonk, NY, USA). Demographic variables such as age and gender distribution of the participants were analyzed using percentage analysis. The paired samples t-test was used for analysis of the pre-intervention and post-intervention values of both outcome measures: the BOT-2 and CPRS. Prior to inferential analysis, the normality of all variables was confirmed using the Shapiro-Wilk test (all p > 0.05), validating the use of parametric testing. The arithmetic mean and standard deviation (SD) were calculated for each outcome measure.

According to Table 2, bimanual training with lower-limb assistance demonstrated a statistically significant improvement in BOT-2 scores in children with ADHD following the intervention. The mean BOT-2 score improved from 32.17 ± 3.35 pre-intervention to 41.11 ± 3.08 post-intervention, with a mean gain of 8.94 points (27.81%), indicating clinically meaningful enhancement in motor coordination and motor proficiency. The paired t-test showed a highly significant difference (t = 13.27, p < 0.001), suggesting improved fine and gross motor coordination, bilateral integration, and overall motor control after the intervention.

**Table 2:** Pre- and post-intervention comparison of BOT-2 scores BOT-2: Bruininks-Oseretsky Test of Motor Proficiency, Second Edition

Scale	Pre value	Post value	t-value	p-value	Interpretation
BOT-2 (n = 18)	32.17 ± 3.35	41.11 ± 3.08	13.27	<0.001	Statistically significant improvement in motor coordination.

According to Table 3, CPRS scores showed a statistically significant reduction following the intervention, indicating decreased ADHD symptom severity as reflected in the total CPRS-R score, particularly in attention and concentration. The mean score decreased from 160.61 ± 25.39 pre-intervention to 105.56 ± 14.42 post-intervention, with a mean reduction of 55.06 points (34.28%), reflecting clinically substantial improvement in ADHD symptoms. The paired t-test demonstrated a highly significant difference (t = 17.29, p < 0.001), suggesting improved attentional control, reduced impulsivity, better activity regulation, and positive effects on cognitive-motor regulatory processes in children with ADHD. This improvement reflects the total CPRS-R score, domain-specific subscale scores were not separately extracted or analyzed in this study, and this is addressed further as a limitation in the Discussion.

**Table 3:** Pre- and post-intervention comparison of Conners' Parent Rating Scale scores ADHD: attention-deficit/hyperactivity disorder

Scale	Pre value	Post value	t-value	p-value	Interpretation
Conners' Parent Rating Scale (n = 18)	160.61 ± 25.39	105.56 ± 14.42	17.29	<0.0001	Statistically significant reduction in ADHD symptom score.

## Discussion

The present study analyzed the effectiveness of bimanual training using lower extremity support for attention, motor coordination, and concentration in children with a diagnosis of ADHD between six and 12 years of age. After a structured intervention of 12 weeks, a significant improvement in both outcome measures was shown, the BOT-2 and the CPRS. The results obtained in this study correspond with the increasing body of evidence that shows that the use of motor-based, non-pharmacological interventions is beneficial for motor and cognitive outcomes in children with ADHD [19]. This age window from 6-12 years is especially important because it is a time of significant maturation of the cortex, motor learning, and neuroplasticity, the period during which therapeutic intervention may have the greatest impact [16]. Liu et al. found a significant positive effect on overall motor proficiency for children and adolescents with ADHD compared to controls (standardized mean difference (SMD) = 1.12, 95% CI (0.63 to 1.61), p < 0.05), and also for fine manual control (SMD = 1.14, 95% CI (0.31 to 1.98), p < 0.05) and bilateral coordination (SMD = 1.54, 95% CI (0.82 to 2.25), p < 0.05) [22]. The present study involved the bimanual protocol, which focuses on the motor abilities across bilateral coordination, manual dexterity, and balance subtests, which have consistently been reported as impaired in children having ADHD, according to Adhvaryu et al. in their systematic review of the use of the BOT to assess motor abilities of children with ADHD [24]. The benefits of the BOT-2 scores in the present study were attributed to the structured, progressive model of bimanual training that incorporated both upper- and lower-limb movements in a coordinated goal-directed manner. Bimanual coordination tasks require interlimb timing, interhemispheric communication, motor planning, and executive control, which are known to be deficient in children with ADHD [15]. Klimkeit et al. reported that children with ADHD showed highly significant differences in timing and in synchronization between both hands in bimanual coordination tasks, which showed a strong correlation with their impaired functioning of neural systems involved in motor planning and executive control [15]. This systematic intervention focused on interlimb coordination and postural stability using various equipment, including the cloud hopscotch mat, arm and foot hopscotch, and Swiss ball therapy, stimulated neural circuits that support bimanual coordination and resulted in the measured changes in BOT-2 scores.

Kleeren et al. found motor-based interventions to generally be effective in children with ADHD, showing maximal improvement in gross motor tasks and tasks involving coordinated limb movement in the Journal of Attention Disorders [23]. The findings of the present study corroborate these findings: Motor coordination and motor proficiency were improved, especially in bilateral coordination, demonstrating the clinical relevance of systematic motor training as a therapeutic strategy in the treatment of pediatric ADHD. The findings from the CPRS scores demonstrated a clinically and statistically significant reduction following the intervention, which is consistent with the findings of Chan et al., who reported that physical activity interventions significantly reduced ADHD-related symptoms, such as inattention and hyperactivity, and concluded that repetitive motor activity could lead to neurobiological changes that facilitate attentional regulation in children with ADHD [25]. Structured play therapy was also proven effective in enhancing attention, decreasing hyperactive behavior, and increasing social interaction in ADHD children compared to control groups by El-Nagger et al. [17]. In the current investigation, the bimanual training protocol employed was play-based and child-centered, using fun and interesting equipment like rainbow sorting tasks, sensory boards, and mini disc cones [18]. The study findings align closely with the findings of Norouzi et al., who explored how neurofeedback training influences bimanual coordination in a sample of 20 children diagnosed with ADHD. Their research indicated substantial progress in both in-phase and anti-phase bimanual coordination protocols after the intervention [26]. However, whereas neurofeedback focuses explicitly on neurological self-regulation, the bimanual motor protocol utilized in this study delivers comparable benefits by leveraging a motor-cognitive participation strategy that offers a more accessible, economically viable, and stimulating alternative for younger populations. Furthermore, the markedly superior outcomes documented in this investigation (a 27.81% advancement in BOT-2 scores alongside a 34.28% decrease in Conners' scores) across an identical 12-week timeframe indicate that task-specific bimanual exercises integrating the lower extremities may yield more comprehensive functional gains than standalone neurofeedback therapy.

A defining characteristic of this intervention was the structured integration of lower-limb exercises into the bimanual training protocol, drawing conceptual inspiration from the HABIT-ILE framework. The HABIT-ILE approach combines bilateral upper extremity tasks with lower extremity mobility to foster comprehensive whole-body coordination, utilizing task-specific practice rooted in goal-directed training and motor learning principles [19]. Although HABIT-ILE has been predominantly standardized for children with cerebral palsy, its foundational tenets-specifically intensive repetition, task specificity, and interlimb coordination-hold direct relevance for children with ADHD, a population that exhibits comparable impairments in motor planning and interlimb synchronization [27]. Furthermore, incorporating vestibular training modalities into the current regimen-such as swing therapy, trampoline exercises, and half-sphere balance board tasks-delivered vital vestibular and proprioceptive feedback. These inputs are well-documented to help regulate attention, postural stability, and physiological arousal in children presenting with ADHD. It is particularly important that children with ADHD demonstrate marked impairments in static balance when subjected to sensory disruptions; they tend to rely disproportionately on visual cues to maintain stability rather than utilizing vestibular and somatosensory feedback [21].

Moreover, this research highlights that managing motor coordination difficulties should be treated as a central pillar of ADHD intervention rather than an incidental issue. This concurs with the conclusions of Watemberg et al., who highlighted the necessity for early detection of motor coordination impairments in children diagnosed with ADHD, noting that targeted physiotherapy regimens significantly enhance both general motor proficiency and day-to-day functional capabilities within this demographic [8]. The current investigation substantiates these recommendations by offering a systematic, evidence-based protocol designed for seamless practical application within outpatient rehabilitation facilities and pediatric physiotherapy clinics.

Strengths, limitations, and recommendations

The present study demonstrated significant improvements in attention, coordination, and concentration among children with ADHD following a structured bimanual training program incorporating lower-limb activities, highlighting its potential as a cost-effective and engaging physiotherapy intervention. The observed improvement reflects changes in the total CPRS-R score; however, domain-specific subscale scores, such as inattention and hyperactivity-impulsivity, were not separately extracted or analyzed in this study, and this issue is further addressed as a limitation. Despite the promising findings, certain limitations should be acknowledged. The study utilized a relatively small sample size and convenience sampling, which may have introduced selection bias and limited the generalizability of the findings. Additionally, the absence of a control group restricted the ability to establish causal relationships. Furthermore, the analysis of only the total CPRS-R score limited the interpretation of which specific behavioral domains were most responsive to the intervention. Future studies should employ larger sample sizes with randomized controlled trial designs to strengthen the evidence base and improve external validity. Long-term follow-up assessments are also recommended to determine the sustainability of treatment effects over time. Moreover, future research should report CPRS-R subscale-level outcomes to provide more clinically meaningful insights and compare bimanual training with other physiotherapy interventions, sensory integration approaches, and pharmacological treatments to identify the most effective management strategies for children with ADHD.

## Conclusions

The present study investigated the efficacy of bimanual training with lower-limb activities on motor coordination and overall behavioral symptoms of ADHD in children, and demonstrated statistically significant improvements in motor proficiency (BOT-2) and overall ADHD symptom severity following the intervention. BOT-2 scores showed notable gains in bilateral coordination, balance, and motor planning, while the reduction in total CPRS-R score reflects a broad improvement in ADHD-related behavior encompassing inattention, hyperactivity, and impulsivity collectively. As domain-specific subscale analysis was not performed in this study, the relative contribution of attention and concentration specifically cannot be isolated from the overall score. This supports the hypothesis that structured bimanual motor training with lower-limb involvement positively influences neural circuits related to motor control and attentional-behavioral regulation broadly. The play-based and progressively challenging activities facilitated neuroplastic changes and enhanced sensory-motor-cognitive integration, suggesting that this intervention serves as a holistic, non-pharmacological, and child-friendly adjunct to existing ADHD management strategies, warranting further investigation through larger randomized controlled trials.
